# On the Treatment of Fracture of the Inferior Maxilla

**Published:** 1873-06

**Authors:** D. R. Silver

**Affiliations:** Sidney, Ohio.


### ARTICLE X.

### *On the Treatment of Fracture of the Inferior Maxilla.*

BY D. R. SILVER, M. D., OF SIDNEY, OHIO.

Fractures of the inferior maxilla are notoriously difficult to treat. This is true, simply because the obvious indication, namely, to keep the fragments in accurate coaptation for a sufficient time for union to take place, is difficult to accomplish. Difficulties exist in the very nature of the office performed by this bone. To take solid food, to swallow liquids, even the saliva; to talk, to cough, or to expectorate, requires in it mobility, unless there be a fixed point of support for it. This support is furnished by the superior maxilla, and, therefore, in the treatment of such fractures the patient is directed to *keep his mouth closed*, a direction wholly superfluous (if the incisors, or some one of them, have not been lost,) because it cannot be accomplished. To such a strait have surgeons been driven, in order to keep the jaw fixed, that food has been introduced through the nares, or through the aperture made by the removal of a sound tooth. Should these measures not be taken, the patient suffers emaciation for want of proper food, or union is delayed by frequent movement of the fragments. All surgeons are aware of the various methods adopted in these troublesome cases. But, so far as I know, none have used and reported that which I devised and found eminently efficient and satisfactory in the following cases:—

Mr. D., aged 30, a laborer, while felling a tree, was struck on the *right* side of the face by a piece of wood, fracturing the inferior maxilla on the *left* side, at a point corresponding with the first molar tooth.

Four weeks after the accident patient presented himself to me for treatment. Examination revealed a transverse fracture at the point before specified, fragments freely moveable, crepitatiou distinct, first molar very loose and carious, pus exuding from a small abscess in the gum at the point of injury, first and second upper molars on the same side lost. Patient has a severe cough, otherwise in good health. Owing to his cough, and the fact that his front teeth, upper and lower, are perfect and in apposition, necessitating movement of the jaw to swallow even liquids, the usual treatment for four weeks had wholly failed.

Under the circumstances, I adopted the following expedient. A competent dentist drew the carious tooth, and was then directed to take an impression of patient's mouth as for artificial upper teeth, the soft wax taking the impression of the upper molars *on both sides*, and spaces where no teeth existed; the lower jaw was now also closed upon the wax until the incisors approached; leaving a space between of barely half an inch, the fracture at the same time being accurately adjusted. The wax being removed, side-pieces were moulded upon it to fit the gums as splints, at the point of fracture A cast was now taken in vulcanized rubber, which answered every indication. We had therefore a *perfect* splint. Placed in the mouth, the jaw secured by a piece of adhesive plaster, the mobile member was found to be alnost immovable, yet the patient could eat, drink, talk, cough and expectorate with comparative comfort. After twenty days union was sufficiently perfect to permit removal of the splint.

The advantage secured by this arrangement are sufficiently obvious, but not the least was this; that *lateral* movement was prevented while the patient slept, and support given to *both* sides of the jaw, securing great comfort to the patient and rapid union of the divided bone.—*Med. & Surg. Reporter.*

